# Utilization of a stem cell‐derived 3D Alzheimer's Disease neurosphere model to investigate the role of ApoE on neuronal/Glial interaction

**DOI:** 10.1002/alz70855_105390

**Published:** 2025-12-24

**Authors:** Christopher Lee, Stefan Wendt, Ada J Lin, Jessica Huang, Haakon B. Nygaard

**Affiliations:** ^1^ University of British Columbia, Vancouver, BC, Canada

## Abstract

**Background:**

Alzheimer's disease (AD) is a chronic neurodegenerative disorder characterized by a progressive deterioration in multiple facets of cognitive function. Genome‐wide association studies have confirmed that the strongest genetic risk factor for AD is polymorphism in the apolipoprotein E encoding gene APOE, with the ε3 allele being considered neutral risk, ε2 and the more recently described APOE ε3 Christchurch mutation conferring protection, and ε4 conferring risk. It is unclear exactly how APOE increases AD risk or hastens AD development, but elucidating these mechanisms is critical in developing a possible therapy. In this work we investigated APOE in an in vitro induced human pluripotent stem cell (hiPSC)‐derived 3D neurosphere model system containing neurons, astrocytes, and microglia to effectively recreate the natural cellular environment.

**Method:**

Neuronal/astrocyte neurospheres were formed from hiPSCs harboring homozygous APOE variants for ε2, ε3, ε4, and ε3christchurch (ε3ch) and matured over a 60‐day period, while hiPSC‐derived microglia for the same isotypes were separately differentiated and applied to spheres. AD‐like pathology was investigated through a chronic treatment of synthetic oligomeric amyloid‐beta treated over 5‐weeks.

**Result:**

Expression of ApoE rose with oligomeric amyloid‐beta treatment, and variation in resistance to chronic amyloid‐beta treatment‐induced functional degeneration was found to mimic natural resistance, with ε2 > ε4 displaying delayed decline of neuronal activity; this was observed both with‐ and without the presence of microglia.

**Conclusion:**

This work provides validation of a protocol for the generation of hiPSC‐derived neurospheres consisting of neurons, astrocytes and microglia. Demonstrating its value as a disease model system for the study of ApoE and AD.

